# Supplementary material for: Cu(II) Binding Increases the Soluble Toxicity of Amyloidogenic Light Chains
Source: Int J Mol Sci. 2022 Jan 16;23(2):950. doi: 10.3390/ijms23020950 (PMC8780072; doi:10.3390/ijms23020950)
Supplement: Supplementary file 1 [file ijms-23-00950-s001.zip › ijms-1530357-supplementary.pdf]

Supplementary material

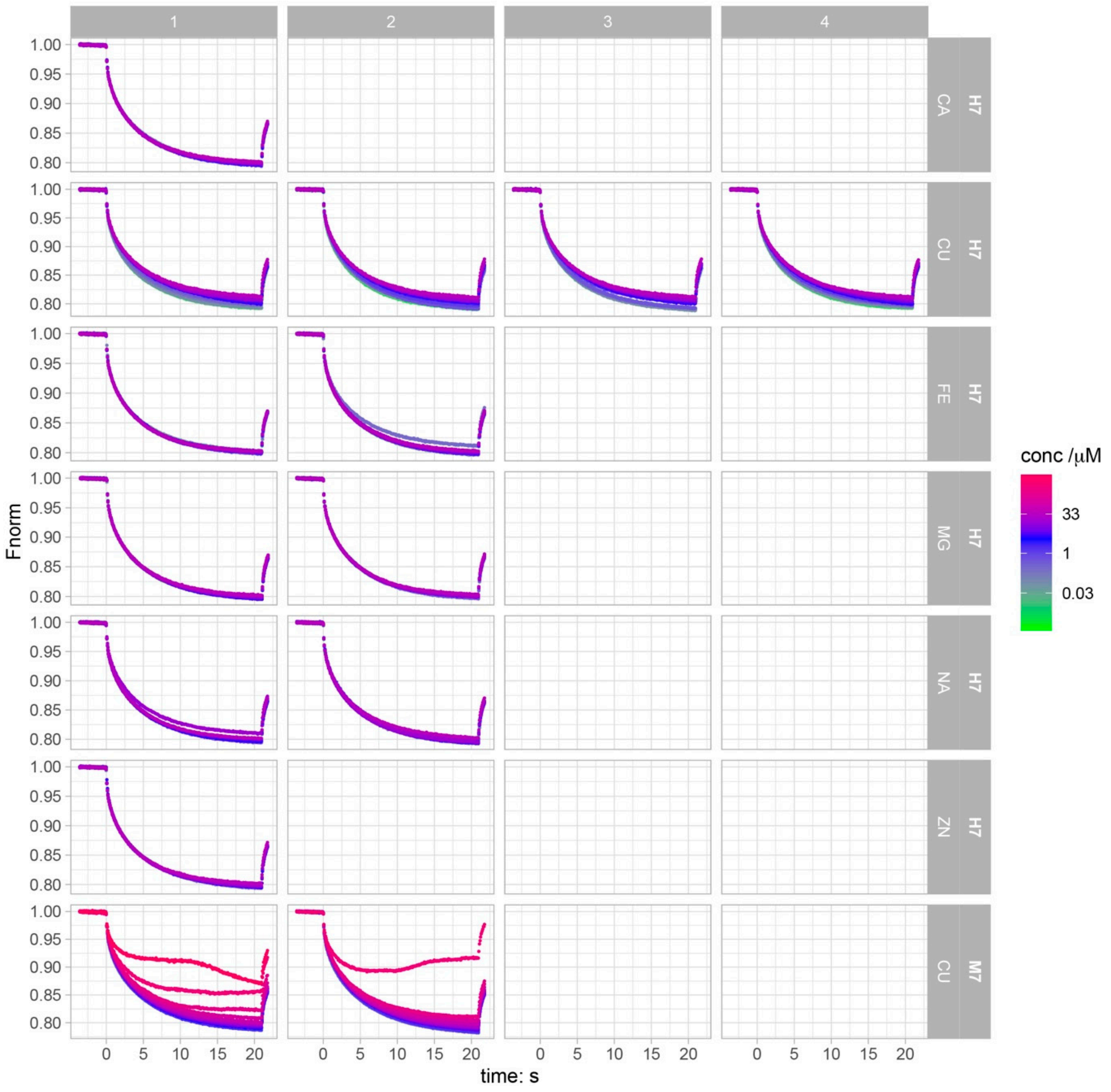

Figure S1. MST thermophoretic curves (MST-TT)

Complete set MST-TT used for the MST titration curves. Plots are organized columns split by experiment number and rows split by light-chain/ion combination. Color-coded as in Figure 1B.

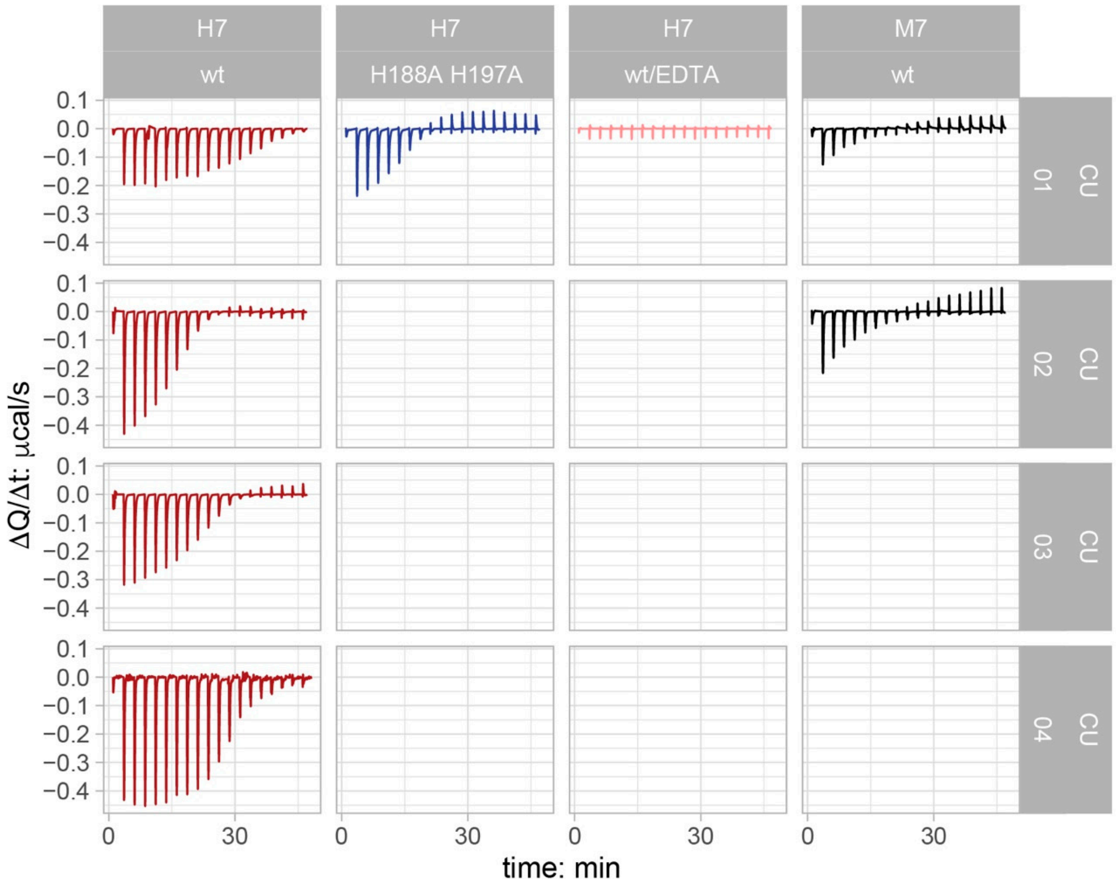

Figure S2. ITC thermograms

Complete set of baseline-corrected ITC thermograms used for the ITC binding isotherms shown in Figure 1C. Plots are organized in columns split by light chain type/EDTA combination and rows split by experiment number.

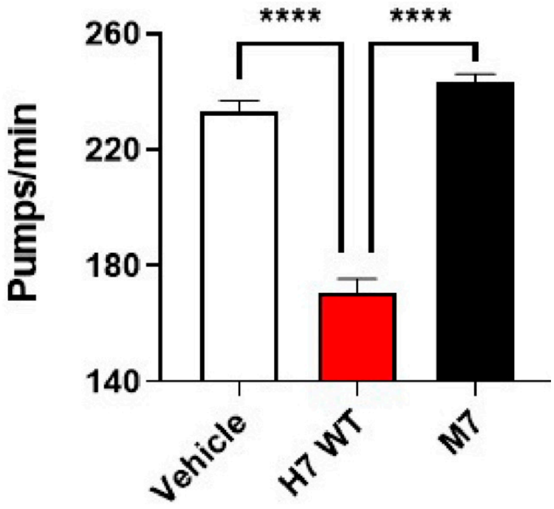

Figure S3. H7 and M7 *in vivo* toxicity

Worms were fed for 2 h with 100 µg/mL H7 WT or M7 LC dissolved in 10 mM PBS pH 7.4. Control worms received vehicles alone (Vehicle). Pharyngeal pumping was determined 24 h after the administration. Each value is the mean ± SE, n = 20. ****p<0.001, one-way ANOVA and Bonferroni's *post hoc* test.
